# Acquired CNS Demyelinating Syndrome in Children Referred to ShirazPediatric Neurology Ward

**Published:** 2014

**Authors:** Soroor INALOO, Saeedeh HAGHBIN, Mehrpoor MORADI, Hassan DASHTI, Nazila SAFARI

**Affiliations:** 1 Neonatal Research Center, Shiraz University of Medical Sciences, Shiraz, Iran; 2 Pediatric Department, Shiraz University of Medical Sciences, Shiraz, Iran; 3 Radiology Department, Shiraz University of Medical Sciences, Shiraz, Iran

**Keywords:** CNS demyelinating syndrome, Multiple sclerosis, Optic neuritis, Acute disseminated encephalomyelitis, Transverse myelitis, Children

## Abstract

**Objective:**

Incidence of CNS acquired demyelinating syndrome (ADS), especially multiple sclerosis (MS) in children, appears to be on the rise worldwide. The objective of this study was to determine prevalence, clinical presentation, neuroimaging features, and prognosis of different types of ADS in Iranian children.

**Materials & Methods:**

During the period 2002-2012, all the patients (aged 1-18 years) with ADS, such as MS, acute disseminated encephalomyelitis (ADEM), optic neurotic (ON), Devic disease, and transverse myelitis (TM), referred to the pediatric neurology ward, Nemazee Hospital, Shiraz University of Medical Sciences, were included in this study. Demographic data, clinical signs and symptoms, past and family history, preclinical findings, clinical course, and outcome were obtained.

**Results:**

We identified 88 patients with ADS in our center. The most prevalent disease was MS with 36.5% (n=32), followed by AEDM 26.1% (n=31), ON 17% (n=13), TM 15.9% (n=14), and Devic disease 4.5% (n=4). MS, ON, TM were more common among females while ADEM was more common in males. Children with ADEM were significantly younger than those with other types of ADS.

Family history was positive in 10% of patients with MS. Previous history of recent infection was considerably seen in cases with ADEM.

Clinical presentation and prognosis in this study was in accordance with those in previous studies on children.

**Conclusion:**

In this study, the most common type of ADS was MS, which was more common in female and older age cases. ADEM was more common in male and younger children. ADEM and ON had the best and Devic disease had the worst prognosis.

## Introduction

Multiple sclerosis (MS), optic neuritis (ON), transverse myelitis (TM), clinically isolated syndrome (CIS), Devic disease, and acute disseminated encephalomyelitis (ADEM) are collectively known as acquired demyelinating syndrome (ADS) (1). 

In children, ADS is rare with unknown prevalence, but its incidence is increasing. Few studies have addressed the incidence of pediatric ADS. A study from Canada showed incidence of 0.9 per 100,000 with 43% MS, 22% ADEM, 23% ON, and 22% TM (2).

MS is the most prevalent of such diseases in adults. It is a chronic debilitating disease that mostly involve young adult. Its prevalence is 75 per 100,000 population in USA and 40 per 100,000 populations in Iran. About 5% of adults who develop MS had their first attack in childhood (3-5). Etiology of ADS is unknown, but genetic and environmental factors are influential in the pathogenesis of the disease (6).

According to literature, most of the studies have been conducted in European countries. Reported data from Asia and, especially on Iranian children are very limited. The aim of this study was to determine the prevalence of different types of ADS, clinical and paraclinical findings, and their outcomes in the studied population.

## Materials & Methods

In this prospective cross-sectional study, we assessed all children aged 1-18 years with criteria of ADS, referred to pediatric neurology ward, Nemazee Hospital, Shiraz, Iran during 2002- 2012. This hospital serves as the referral center in south of Iran. Diagnosis of all types of ADS (MS, ADEM, ON, TM, and Devic disease) was based on the consensus definition for pediatric ADS (5,7). Inclusion and exclusion criteria are summarized in Table 1 (8).

A questionnaire on demographic features, clinical sign and symptoms, recent history of infection and vaccination, family history of ADS, paraclinical findings (laboratory, brain MRI), and clinical course and prognosis, was filled in for each patients based on respective medical records.

The collected data were analyzed by SPSS software (version 19). Inferential statistics including correlation and Mann-Whitney test and descriptive statistics, including central and dispersion tendencies were used. P-value less than 0.05 was considered statistically significant. Informed consents were obtained from patients or their parents. The study design was approved by the Ethics Committee of Shiraz University of Medical Sciences.

## Results

We identified 88 patients with ADS in our study. Annual incidence of ADS in Fars province is about 0.19 per 100.000 persons consisting of 59.1% females and 40.9% males. The most prevalent disease was MS with 36.5% (n=32), followed by ADEM 26.1% (n=23), ON 17% (n=15), TM 15.9% (n=14), and Devic disease 4.5% (n=4). The age of patients ranged between 1 and 18 years with mean of 12±4.8 years, and the mean age of onset of symptoms was 11±4.71 years. The mean age for Devic cases was the highest (16.3 years), and for ADEM was the lowest (7.6 years) (Table 2).

Female to male ratio in MS cases was 3/1, in ADEM 1/2, and in ON 2/1. The Most common presenting feature was visual impairment (51.1%), followed by paraplegia (37.5%), ataxia (28.4%), and impaired consciousness (23.9%) (Table 3).

Most frequent season of presentation of patients was autumn (31.8) and the least was in spring (11.4%). It was observed that 41 cases of ADS (46.6%) had a history of recent infection, of whom patients with ADEM showed the highest rate of positive recent infection (73%). Positive family history of demyelination disease was about 10%, all being with MS (Table 2). 

Most common presenting features in patients with MS were visual impairment, ataxia, and paresthesia, and in ADEM cases were impaired level of consciousness, paraplegia, and seizure (Table 3). Eighty (80%) patients had brain and spinal cord MRI. The most common findings of the brain imaging are shown in (Table 4).

Visual evoked potential (VEP) was done in 66 patients, of whom 29 (33%) were abnormal, mostly in those with ON and MS. Fifty-nine patients underwent lumbar puncture, of whom 26 (30%) had mild pleocytosis with increased protein.

Vasculitis serology work-up was done for 66 patients, of whom 4 patients were found to be positive for antinuclear antibody (ANA), all of them were in MS group. Of 32 patients with MS, 28 received follow-up, and all had EDSS (expanded disability status scale) score below 4, indicating minimal disability. Recurrent disease occurred in 22 (25%) patients consisting of 17 with MS, 2 with ON, 1 with ADEM, and 2 with Devic disease. In cases with ADEM, 1 patient expired, 2 patients had mild disability in the follow-up, and others had complete recovery. Among 15 patients with ON, 3 had mild visual impairment, 14 had complete recovery, and 1 developed MS after 3 years. Seven (50%) patients with TM had complete recovery, 2 had mild motor disability, 3 had severe motor disability (complete paralysis), and 3 had mild sphincter dysfunction. In follow-up, from 4 patients with Devic disease, 2 had multiple recurrences, 1 had blindness with paralysis, 1 patient developed blindness, and 1 had paralysis. 

**Table 1:** Summarized inclusion definitions for CNS acquired demyelinating syndromes

**Condition**	**Definition**
Acute disseminated encephalomyelitis (ADEM)	(1) A polysymptomatic clinical event with acute/subacute onset that must include encephalopathy (behavioral change or altered consciousness). (2) MRI brain shows multifocal lesions.
Clinically isolated syndrome (CIS)	A first acute-clinical episode of CNS symptoms which may either be monofocal or multifocal, but does not include encephalopathy (except in brainstem syndromes). The MRI will show white matter demyelination. These include:1. Transverse myelitis (TM): weakness and/or numbness of both legs +/- arms, usually with maximal deficits 1 week after symptom onset supported by demyelination on MRI spine.2. Optic neuritis: Acute or subacute loss of vision and ≥1 of: relative afferent pupillary defect (unilateral cases), visual field deficit or scotoma, impaired colour vision, optic disc edema, or abnormal visual evoked potentials. MRI is not necessary for diagnosis.3. Other CIS: Brainstem, cerebellar, and/or hemispheric dysfunction, supported by demyelination on MRI.
Neuromyelitis optica (NMO)	Must have: i. Optic neuritis and ii. Acute myelitis.Must have: iii. Spinal MRI lesion extends over three or more segments or iv. Aquaporin-4 antibody testing is positive.The brain MRI may be abnormal but must not meet Multiple Sclerosis MRI diagnosis criteria.
Exclusion criteria	1. Leukodystrophies (e.g. metachromatic leukodystrophy, adrenoleukodystrophy) or mitochondrial disease.2. Proven CNS infection (e.g. viral encephalitis, bacterial meningitis, herpes simplex encephalitis, Lyme disease, HIV).3. Radiation/chemotherapy associated white matter damage.4. Condition fulfilling criteria for CNS connective tissue disease e.g. lupus, vasculitis. The sole presence of antibodies associated with CNS connective tissue or autoimmune diseases was not sufficient for exclusion.

**Table 2:** Demographic characteristic of pediatric ADS cases

	**MS**	**ADEM**	**ON**	**TM**	**Devic**	**Total**
**Frequency **	32	23	15	14	4	88
**Mean age**	13.8	7.6	11.6	13.2	16.3	12
**Female/ male**	24/8	15-Aug	5-Oct	6-Aug	2-Feb	52/36
**Positive family history**	8	0	0	0	0	8
**History of recent infection **	Jul-32	17/23	15-May	14-Sep	4-Mar	41/88
	-21%	-73%	-33%	-64%	-75%	-46%

**Table 3:** Frequency of the first clinical symptoms in patients with ADS

**Disease**	**Device**	**ADEM**	**MS**	**ON**	**TM**
**Symptoms**					
**Ataxia**	2	5	12	4	2
**Speech disorders**	0	3	4	0	0
**Hemiplegia**	0	0	7	0	1
**Decreased LOC**	0	15	3	3	0
**Convulsion**	1	7	1	1	0
**Visual disturbances**	4	4	20	15	2
**Paraplegia**	3	9	7	1	13

**Table 4:** MRI findings in patients with ADS

**MRI finding (site of involvement)**	**Number**	**Percent**
Croups callosum	6	6.8
Brain stem	9	10.2
Spinal cord	29	32.9
Cerebellum	10	11.4
Deep white matter	43	48.9
Internal capsule	5	5.7
Basal ganglia	2	2.3
Thalamus	6	6.8
Optic nerve	6	6.8
Normal MRI	8	9.1

## Discussion

According to the results of the present study, annual incidence of ADS in Fars province is about 0.19 per 100.000 persons. It is low in comparison with the rate in Canadian study, which was 0.9 per 100.000 children and in South California, which was 1.66 per 100.000 persons (1,2). Probably, all children with ADS were not referred to our center and some of them had been followed by adult neurologist or ophthalmologist. MS was the most common type of ADS in the present study (32%), which is consistent with the rates reported from UK, Ireland, and US (about 31%) (1,8). However, ON was the most common among Canadian children 23% (2). 

Female to male ratio in this study was 1.4:1, consistent with that of Canadian study (1.09:1) and UK (1.3:1), but different from a study by Etemadifar in Iran (4.5:1) (12). Female to male ratio in patients over 10 years old was 1.6:1 and it was 1.1:1 in those under 10 years. In agreement with other studies, it was found that MS, ON, and TM were more common in females and ADEM was more common in males (1).

Similar to other studies, we found that MS is polyphasic but ADEM is mostly monophasic and more common in males, and patients are under 10 years of age (1,2,7,9). 

Most common season of presentation of ADS in the present study was autumn, but it was spring in the studies by Jin et al. and Ghobai et al. (10,11).

Regarding positive family history, the rate of MS in children was reported to be 0-21% (12-15) and it was 10% in the present study and 9% in another study from Iran (12). History of recent infection was positive in 46.5% of our patients, mostly in ADEM cases. 

In this study, the most common presenting feature of MS was visual impairment followed by ataxia. Decreased level of consciousness, seizure, and paraplegia, were the most frequent features in ADEM cases, consistent with other studies (12,16-18). Autoantibody such as antinuclear antibody (ANA) was positive in 4 (12%) patients with MS, other studies reported it to be 22.5% to 26.7% in adult population with MS (19,20).

According to clinical course and outcome, EDSS score of MS patients in our study was less than 4, which has been also reported in some previous studies (12,13).

Prognosis of ON in our patients was good and 82% had complete recovery, while only 1 patient developed MS in follow-up, similar to other reports in children (21,22). 

As revealed in the present study, 50% of TM patients had complete recovery, 14% had mild disability, 20% were wheel chair dependent and 20% developed persistent bladder dysfunction. Similarly, in a study by Thomas, 16% were wheel chair dependent and 22% had bladder dysfunction (23).

Given the increasing rate of ADS in Iranian children, developing a registration system for such patients is suggested in order to obtain more accurate information and consequently improve diagnosis, management, and prognosis of ADS.
